# Supplementary material for: Land use is a stronger determinant of ecological network complexity than the number of trophic levels
Source: PLoS One. 2024 Feb 9;19(2):e0295377. doi: 10.1371/journal.pone.0295377 (PMC10857743; doi:10.1371/journal.pone.0295377)
Supplement: Attachment — Submitted filename: Response to Reviewers.docx [file pone.0295377.s001.docx]

Response to reviewers:

Thank you very much for all comments made by the reviewers. They helped us to improve the quality and clarity of the manuscript. Our responses are in normal letter following each of the comments highlighted in bold type lettering. Line numbers in the responses correspond to lines in the “Manuscript” file.

Please submit your revised manuscript by Feb 09 2023 11:59PM. If you will need more time than this to complete your revisions, please reply to this message or contact the journal office at [plosone@plos.org](mailto:plosone@plos.org). When you're ready to submit your revision, log on to <https://www.editorialmanager.com/pone/> and select the 'Submissions Needing Revision' folder to locate your manuscript file.

Please include the following items when submitting your revised manuscript:

- A rebuttal letter that responds to each point raised by the academic editor and reviewer(s). You should upload this letter as a separate file labeled 'Response to Reviewers'.
- A marked-up copy of your manuscript that highlights changes made to the original version. You should upload this as a separate file labeled 'Revised Manuscript with Track Changes'.
- An unmarked version of your revised paper without tracked changes. You should upload this as a separate file labeled 'Manuscript'.

If you would like to make changes to your financial disclosure, please include your updated statement in your cover letter. Guidelines for resubmitting your figure files are available below the reviewer comments at the end of this letter.

If applicable, we recommend that you deposit your laboratory protocols in protocols.io to enhance the reproducibility of your results. Protocols.io assigns your protocol its own identifier (DOI) so that it can be cited independently in the future. For instructions see: [https://journals.plos.org/plosone/s/submission-guidelines#loc-laboratory-protocols](https://nam10.safelinks.protection.outlook.com/?url=https%3A%2F%2Fjournals.plos.org%2Fplosone%2Fs%2Fsubmission-guidelines%23loc-laboratory-protocols&data=05%7C01%7Caamarillo%40javeriana.edu.co%7C8ef14515f8a046cc6d7f08dae7b95ed2%7Cdaf7990e8a3f409c9b762a5475098000%7C0%7C0%7C638077080609422994%7CUnknown%7CTWFpbGZsb3d8eyJWIjoiMC4wLjAwMDAiLCJQIjoiV2luMzIiLCJBTiI6Ik1haWwiLCJXVCI6Mn0%3D%7C3000%7C%7C%7C&sdata=w7%2F4Au5NdGCAfX9lsWh8xG57vRpLorssD%2BA%2Fwhchui8%3D&reserved=0). Additionally, PLOS ONE offers an option for publishing peer-reviewed Lab Protocol articles, which describe protocols hosted on protocols.io. Read more information on sharing protocols at [https://plos.org/protocols?utm_medium=editorial-email&utm_source=authorletters&utm_campaign=protocols](https://nam10.safelinks.protection.outlook.com/?url=https%3A%2F%2Fplos.org%2Fprotocols%3Futm_medium%3Deditorial-email%26utm_source%3Dauthorletters%26utm_campaign%3Dprotocols&data=05%7C01%7Caamarillo%40javeriana.edu.co%7C8ef14515f8a046cc6d7f08dae7b95ed2%7Cdaf7990e8a3f409c9b762a5475098000%7C0%7C0%7C638077080609422994%7CUnknown%7CTWFpbGZsb3d8eyJWIjoiMC4wLjAwMDAiLCJQIjoiV2luMzIiLCJBTiI6Ik1haWwiLCJXVCI6Mn0%3D%7C3000%7C%7C%7C&sdata=GiNssl970Bxhq3zLfi0iGOlp3qlOgSe1wwdhSbmDgJ4%3D&reserved=0).

When submitting your revision, we need you to address these additional requirements.

1. Please ensure that your manuscript meets PLOS ONE's style requirements, including those for file naming. The PLOS ONE style templates can be found at

<https://journals.plos.org/plosone/s/file?id=wjVg/PLOSOne_formatting_sample_main_body.pdf> and

[https://journals.plos.org/plosone/s/fileid=ba62/PLOSOne_formatting_sample_title_authors_affiliations.pdf.2](https://nam10.safelinks.protection.outlook.com/?url=https%3A%2F%2Fjournals.plos.org%2Fplosone%2Fs%2Ffileid%3Dba62%2FPLOSOne_formatting_sample_title_authors_affiliations.pdf.2&data=05%7C01%7Caamarillo%40javeriana.edu.co%7C8ef14515f8a046cc6d7f08dae7b95ed2%7Cdaf7990e8a3f409c9b762a5475098000%7C0%7C0%7C638077080609422994%7CUnknown%7CTWFpbGZsb3d8eyJWIjoiMC4wLjAwMDAiLCJQIjoiV2luMzIiLCJBTiI6Ik1haWwiLCJXVCI6Mn0%3D%7C3000%7C%7C%7C&sdata=Hrx3jEx55vicdNjZ54GCvm1pdeccPxz1NJaVKPCAUhE%3D&reserved=0).

2. We note that the grant information you provided in the ‘Funding Information’ and ‘Financial Disclosure’ sections do not match.

When you resubmit, please ensure that you provide the correct grant numbers for the awards you received for your study in the ‘Funding Information’ section.

3. Thank you for stating the following financial disclosure:

“-HWR: IDIPI-104: Research Institute of the Escuela Superior Politécnica de Chimborazo (ESPOCH), Riobamba, Ecuador, <https://www.espoch.edu.ec/> NO

ARAS: ID00008226; Pontificia Universidad Javeriana in Bogotá, Colombia. <https://www.javeriana.edu.co/inicio> NO”

Please state what role the funders took in the study.  If the funders had no role, please state: ""The funders had no role in study design, data collection and analysis, decision to publish, or preparation of the manuscript.""

If this statement is not correct you must amend it as needed.

Please include this amended Role of Funder statement in your cover letter; we will change the online submission form on your behalf.

4. We note that you have indicated that data from this study are available upon request. PLOS only allows data to be available upon request if there are legal or ethical restrictions on sharing data publicly. For more information on unacceptable data access restrictions, please see <http://journals.plos.org/plosone/s/data-availability#loc-unacceptable-data-access-restrictions>.

In your revised cover letter, please address the following prompts:

a) If there are ethical or legal restrictions on sharing a de-identified data set, please explain them in detail (e.g., data contain potentially sensitive information, data are owned by a third-party organization, etc.) and who has imposed them (e.g., an ethics committee). Please also provide contact information for a data access committee, ethics committee, or other institutional body to which data requests may be sent.

b) If there are no restrictions, please upload the minimal anonymized data set necessary to replicate your study findings as either Supporting Information files or to a stable, public repository and provide us with the relevant URLs, DOIs, or accession numbers. For a list of acceptable repositories, please see <http://journals.plos.org/plosone/s/data-availability#loc-recommended-repositories>.

We will update your Data Availability statement on your behalf to reflect the information you provide.

**5. Please amend either the abstract on the online submission form (via Edit Submission) or the abstract in the manuscript so that they are identical.**

Response: The abstract in the online submission form has been corrected, and it is identical to the abstract in the manuscript.

6. **Please include your full ethics statement in the ‘Methods’ section of your manuscript file. In your statement, please include the full name of the IRB or ethics committee who approved or waived your study, as well as whether or not you obtained informed written or verbal consent. If consent was waived for your study, please include this information in your statement as well**.

Response: We completed the ethics statement in the Methods section, lines 180-187

**7. Please include captions for your Supporting Information files at the end of your manuscript, and update any in-text citations to match accordingly. Please see our Supporting Information guidelines for more information:** [**http://journals.plos.org/plosone/s/supporting-information**](http://journals.plos.org/plosone/s/supporting-information)**.**

Response: We have not included supporting information in this revised manuscript

Reviewers' comments:

Reviewer's Responses to Questions

**Comments to the Author**

1. Is the manuscript technically sound, and do the data support the conclusions?

The manuscript must describe a technically sound piece of scientific research with data that supports the conclusions. Experiments must have been conducted rigorously, with appropriate controls, replication, and sample sizes. The conclusions must be drawn appropriately based on the data presented.

Reviewer #1: Partly

Reviewer #2: No

Reviewer #3: Partly

2. Has the statistical analysis been performed appropriately and rigorously?

Reviewer #1: Yes

Reviewer #2: No

Reviewer #3: Yes

3. Have the authors made all data underlying the findings in their manuscript fully available?

The [PLOS Data policy](https://nam10.safelinks.protection.outlook.com/?url=http%3A%2F%2Fwww.plosone.org%2Fstatic%2Fpolicies.action%23sharing&data=05%7C01%7Caamarillo%40javeriana.edu.co%7C8ef14515f8a046cc6d7f08dae7b95ed2%7Cdaf7990e8a3f409c9b762a5475098000%7C0%7C0%7C638077080609422994%7CUnknown%7CTWFpbGZsb3d8eyJWIjoiMC4wLjAwMDAiLCJQIjoiV2luMzIiLCJBTiI6Ik1haWwiLCJXVCI6Mn0%3D%7C3000%7C%7C%7C&sdata=13zxx4ou%2F8zkBWX%2BqJwsjCQKdxbvQl5tiLd69%2FtkByo%3D&reserved=0) requires authors to make all data underlying the findings described in their manuscript fully available without restriction, with rare exception (please refer to the Data Availability Statement in the manuscript PDF file). The data should be provided as part of the manuscript or its supporting information, or deposited to a public repository. For example, in addition to summary statistics, the data points behind means, medians and variance measures should be available. If there are restrictions on publicly sharing data—e.g. participant privacy or use of data from a third party—those must be specified.

Reviewer #1: Yes

Reviewer #2: No

Reviewer #3: Yes

4. Is the manuscript presented in an intelligible fashion and written in standard English?

PLOS ONE does not copyedit accepted manuscripts, so the language in submitted articles must be clear, correct, and unambiguous. Any typographical or grammatical errors should be corrected at revision, so please note any specific errors here.

Reviewer #1: Yes

Reviewer #2: Yes

Reviewer #3: Yes

**5. Review Comments to the Author**

Please use the space provided to explain your answers to the questions above. You may also include additional comments for the author, including concerns about dual publication, research ethics, or publication ethics. (Please upload your review as an attachment if it exceeds 20,000 characters)

**Reviewer #1:** RE. Review of PONE-D-22-17757, “Land use is more of a determinant than community size of ecological network complexity of arthropods associated with exotic legumes in the Galapagos, Ecuador”

**The manuscript, submitted to PLOS ONE for review, details an experiment in which legume seedpods were collected from five legume species found in Ecuador at sites where the land is used in different ways. The seedpods were held in laboratory conditions and arthropods using the seedpods were collected. The authors then conducted analyses to determine if the complexity of the communities associated with the seedpods (number of trophic levels, referred to as ‘community size’) or if the land use type where the seedpods were collected had a greater impact on the interactions within each community network. The research was conducted to fill knowledge gaps in the field of ecological network analysis, particularly with regard to interactions between insect herbivores and their natural enemies and the effects of human interference on interaction networks**.

**The authors clearly and concisely describe the current status of research on ecological networks in relation to human interference, especially invasive species. Thus, the Introduction provides ample justification for the work. Although some specific details are missing or are not clearly explained in the Methods (e.g., sampling and total number of sites sampled, please see specific comments below), the work done and the analyses conducted seem sound (although I am not an expert on these aspects of community ecology). The contribution(s) and findings of the work described in the manuscript are addressed in the Discussion and further areas of inquiry are highlighted. Overall, the work is of interest to a broad audience, spanning biologists, ecologists, entomologists, and likely policy analysts and will contribute to valuable discourse in this area of study. I support publishing the manuscript following revisions to clarify the experimental design and the presentation of some of the results.**

Response: We appreciate very much the comments made by reviewer. They helped us to produce a better and more consistent manuscript. We addressed all comments, and the responses below explain the changes we made. We read the comments made by the three reviewers and revised some sections of the manuscript. Specifically, we made changes to the Experimental Design and Results sections to improve the consistency among different sections of the manuscript, as was also suggested by Reviewer #3.

Below are our responses to the comments of Reviewers

**Reviewer** #1:

**Specific Comments by Line Number:
L24. The term “community size” is somewhat misleading. In general, community size is used to describe the number of individuals in a population and perhaps the geographic area occupied by a given community. Here, the authors use “community size” to refer to the number of trophic levels in the communities studied (tri-tropic vs. >3 trophic levels). Perhaps ‘trophic complexity’ or a similar descriptor could be used in place of “community size” throughout the manuscript, so that the factors being studied are more clear to the reader?**

Response: Thank you very much for the comment. We considered this term and agree that it could be misleading to the readers. Therefore, we changed “community size” to “number of trophic levels”. We made this change throughout the manuscript. It is closer to what we intended to express.

**L33-35. Should this sentence read: “…and no significant effect of community size on connectance, diversity, or interaction evenness.”?**

Response: Yes, you are right. Thank you very much. We changed the sentence by eliminating the parenthesis.

**L40-46. This paragraph seems out of place in the Abstract and should be incorporated into the previous paragraph or deleted.**

Response: Thank you very much again. We deleted the paragraph because it was not necessary. **L119-120. The final sentence of the Introduction is a statement of Results, that may be better left to the Discussion.**

Response: Thank you very much. We deleted the sentence

**L134. Why was sampling only conducted in one year? Would the authors expect to see differences between years that could impact the outcome of the study and the conclusions drawn from the analyses?**

Response: Yes, it was. As stated the Methods section, the sampling for this study was conducted at a specific time and in a certain geographical area. The sampling was performed during the year the project was founded; consequently, legal permits were issued for only that period and sampling site. Our research was conducted in a biosphere reserve and a natural park in Ecuador where permits for collecting biodiversity samples are very strict and specify the time and space of sampling. It has been shown that interaction networks change over time, even on very small scales, such as in a matter of days (Bascompte, 2014, and references there in). Thus, it would have been bold, to say the least, to extend our conclusion to other times throughout the year. In addition, this was not the main objective of our study. We did not intend to make comparisons or to show how the interaction networks change over time. This could be the topic of a future study. We understand that it would be desirable for the conclusions of a study to have broader implications; however, they need to reflect the research and be consistent with the data collected to address the study aims. Our research question explored the effect of land use and the number of trophic levels in the interactions; our conclusions can be extended to similar settings but not to comparisons over time.

**L135. Of the four vegetation zones on the island, were all of the deciduous forest areas within the humid zone? As the zones are defined in L125-126, please define which zones the samples were collected in.**

Response: Thank you for the question. As mentioned in the manuscript, the study was conducted in the arid zone. Based on the reviewer’s question, we reviewed the paragraph and found it was unclear, so we changed some sentences in this section.
 **L136-138. How many sites of each land use type were sampled and how many sites were sampled in total? Ten total sites divided by 4 land use types is an inadequate number of sites for rigorous conclusions to be drawn from. Please clarify this aspect of the experimental design.**

Response: We revised the Methods section. We hope it is now clear.

**L138-141. Why collect seeds from five legume species and not just focus on one legume species (or on one endemic species and one invasive species)? Were all five species available at all of the sites that were sampled? Could differences in plant diversity at the sites have impacted the analyses and subsequent conclusions of the study? Sampling intensity is used as a covariate and does not appear to have had a major effect, but plant diversity effects are not directly addressed. Please clarify these aspects of the experimental design.**

Responses:

**First question Why collect seeds from five legume species and not just focus on one legume species (or on one endemic species and one invasive species)?**

Indeed, the questions the reviewer ask would be very interesting to solve on other studies such as those the reviewer states: comparing communities associated to an exotic and a native species, even comparing the same communities among several years, islands, etc. However, that was not the focus of this study. Communities are structured according with their history of change and as we wrote before, that was not the focus of our study. We did what we did because the richness, diversity and interactions of communities of insects associated to a plant are not just depending of a specific plant and its insects. This specific interaction also depends of factors such as other plants that are used by the same insects and of the abundance of the different hosts in a specific land use, if the plant is in addition to exotic, invasive, etc. Exotic and invasive species respond different to community assemblage. In our study there was an invasive species plant (*L. leucocephala*), and the other species are considered as exotic (not invasive species) in the archipelago.

**Second question: Were all five species available at all of the sites that were sampled?**

Response: Table 2, figure 1 show this information. All five species were not all in the sampling sites. In order to gain clarity, we added a sentence and a citation to the table 1 in the methods section (Lines 152-162). We also modified table 1 to show the interactions among animals, plants, and the land use they were found.

**Third question: Could differences in plant diversity at the sites have impacted the analyses and subsequent conclusions of the study? Sampling intensity is used as a covariate and does not appear to have had a major effect, but plant diversity effects are not directly addressed. Please clarify these aspects of the experimental design.**

Response: In fact, it could. That is why we used the sampling intensity index to account for the variation in plants and animals’ richness in our analyses. The sampling intensity index is calculated having in account the number of interactions (abundance of interactions), the number of species of plants (diversity) and the number of species of animals (diversity), and it was calculated for each land use. That is how the metrics works. Lines 213-218 from the data analysis section explained this. However, in order to gain more clarity, we added some additional information in this section (lines 213-218). On the other hand, as much as we say plant diversity could have an impact, insect diversity could too.

**L173. Use “Chi-square”**

Response: Response: We modified the text, as suggested.

**L213-223. With regard to formatting, the paragraph beginning on L213 and the paragraph beginning on L223 should be melded into one paragraph, with the Figure legend/title and Table 2 appearing at the end of the text for that new paragraph. This issue arises from the formatting requirements of the journal and is not the fault of the authors, but does make the text of the Results difficult to follow.**

Response: We changed accordingly. We could changed back if the formatting requirements of the journal say so.

**L215-222. In the title for Figure 1, I believe the colour blue correspond to arthropods that eat plants (but not plant seeds). If this is correct, then ‘herbivore’ would be a better descriptor than ‘phytophagous.’ The final sentence in the figure title could be revised to read: “Lines connecting plants and arthropods represent observed interactions, where broader lines represent interactions that occur with high frequency.”**

Response: Phythophagus is a synonym of herbivorous, so we left the term “phytophagous”. We did not change the final sentence of the explanation of the figure as suggested by the reviewer because it does not clearly describe the connections. We left the description as it was previously.

**L223-224. Table 2 provides a nice summary of the species found in the study and the land use areas where they were found, however, it does not classify or indicate which species were found in PSP communities and which were found in PSPP communities as this sentence indicates. Please revise to ensure the Table is being used and referred to accurately.**

Response: Thank you very much. We changed Table 1 to clarify the interactions between the species on the land use areas and for different number of trophic levels. We also improved the accuracy of the description of the table. Table 2 is no longer necessary.

**L227. Is the F-value correct? Please double check.**

Response: Thank you very much. We changed from, 34,5 to 34.5.

**References (starting L307): The manuscript and concepts therein have been thoroughly researched. However, the authors cite 87 sources, which is quite excessive for a research paper. If possible, the number of references should be reduced.**

Response: We revised the references in the manuscript and retained those we believe are necessary
**Abbreviations are not used in excess in the manuscript, but some abbreviations used in the text are not necessary. For example, the terms ‘interaction evenness’ and ‘interaction strength asymmetry’ could be used in the text rather than the abbreviations IE and ISA, respectively. Doing so would help readers to more easily keep track of which ecological measures are being analyzed, especially readers that are more familiar with the arthropod communities than with these ecological concepts.**

Response: We made changes in some sections

**Reviewer #2:**

**The authors collected 250 grams of seeds from 5 legume tree species at 10 sites that represented 4 land-use types from within the arid zone of Santa Cruz Island, Galapagos, over 2 months in 2018 and reared the associated insects. A number of community metrics were calculated at two community scales, one including all plant and insect species and one including all species except predators. The effect of land-use types on these parameters was assessed using GLM.

One of my first impressions of the article was that it is based on very little sampling over a very short time period compared to other similar studies. However, I have to admit that very many insect individuals were reared, so maybe there is sufficient power to detect some trends.**

Response: We modified the Methods section to make it clearer.

**Some of the main results of the study were a series of community indices, but there seems to be no basis upon which to decide whether the values of these indices are** **‘significant’ or what 95% confidence limits are for them. I presume that indices such as these could be calculated for even a very few samples so it is hard to evaluate how seriously to take them. I would think there is some way to estimate confidence, for instance by the comparison to a null value through a bootstrapping process. Approaches like this are available through the ‘econullnetr’ R package.**

Response: Thank you very much for your comments. We performed significance tests of the metrics, contrasting the observed values with those of the null models and found that all of them were significant. We added this information in the manuscript in the Methods section (lines 209-211) and the Results section (lines 233-235, table 2)

**Other results concern trends associated with the landscape types. I found these to be frustrating though since there was no attempt to correct these effects for host plant species. I would think that host plant is an important driver of insect diversity in a study like this, and while it was clear that the composition of the host plants varied among the site categories from the data tables, there was no quantification of which species predominated at which types of sites. It is not hard to imagine that the trends being ascribed to ‘land-use’ had alot (or at least something) to do with the host-plants present at these sites. I think that an analysis taking this into account would be necessary to properly interpret the results.**

Response: Thank you for the comment. First, we differentiated land use types, not landscape types. This has been consistently stated throughout the manuscript. Land use and landscape are different aspects of land characterization. Second, as stated in the Methods section, we used the sampling intensity index, which accounts for the diversity of plants, animals, and interactions in the algorithm, thus controlling (or correcting) for the variation in plants and animals as well. We agree with the reviewer’s view of the bottom-up regulation of communities in which plants determine the richness of herbivores and higher trophic levels. However, from a top-down perspective, natural enemies and herbivores also play an important role in structuring communities.

**Related to this problem is that this paper is almost completely devoid of natural history. A reader would have no idea which herbivore species is feeding on which plant species, which parasitoid species is feeding on which herbivore species or which predators are present on which plants. Some of the linkages can be figured out from the tables but not most. I really miss these kinds of details and think it is a lost opportunity to ignore them; without them the paper feels very dry and technical.**

Response: Response: Thank you for the comments. We don’t think this is a “related problem”. The previous comment was about how to control for variation in plant diversity and this comment suggests adding information regarding which species are related to each other. To include more natural history, we redesigned Table 1 to show the plant-insect interactions as well. We do not see a problem with the manuscript having a dry and technical tone. We apologize if this was frustrating. We hope the information we added meets the requirements for adding the natural history of the species involved in the networks.


**So – to summarize I feel that the paper as it stands feels like a preliminary study from a limited dataset and provides analyses that are very hard to have confidence in.**

Response: To summarize, we responded to every comment before and provided evidence by using the appropriate metrics and concepts to describe the pattern we found. We considered the comments of all three reviewers to improve the clarity of the manuscript. We hope this addresses the concerns of the reviewer, and that way he/she does not feel frustrated again.
**I have some more minor comments below:
Abstract: the abstract is too long and should not contain statistical details.**
Response: The abstract is within the word count established by the journal

**L40. There seems to be some repeating of material here.**
Response: Thank you for your comment. In deed the paragraph was repeated, so we deleted it.

**L97. It would be good to explain the difference between exotic and invasive here**

Response: We did not find it necessary, nor did the other two reviewers. Therefore, we did not change this sentence.

**L112. I think you mean ‘dispersal’ not ‘dispersion’:**

Response: We mean dispersion: “*The spatial distribution of individual organisms within a local population*” Dispersal is “*the movement of organisms away from their point of origin* (Lomolino et al., 2006). Or according to Nathan (2001, Encyclopedia of biodiversity) dispersion “*refers to the fine-scale spatial distribution pattern of organisms*” and dispersal is “*the movements of disseminules away from their parent source*”. The references cited support the idea of dispersion, not dispersal, which is a biogeographical process on a larger scale.

**L134. ‘season’ not ‘seasons’:**

Response. You are right. We corrected the word.

**L137. Saying ‘including’ in this context is vague. You must list all of the landscape types sampled, along with a description of each and an indication of how many sites of each. From what is presented it looks like there were 4 site classifications and 10 sites so presumably 2 or 3 sites of each landscape type (which seems like very low replication) but it was not clear enough.**

Response: You are right. It is not “including”. Those land use types were the types that were sampled. We corrected the paragraph to make it clearer and added a description of each one (See lines 147 and 151).

**L155. ‘Directorate’ not ‘Direction’**

Response: Thank you very much. We implemented the suggested change.

**L164-166. I don’t understand the rationale between using two ‘community sizes’ for the network analysis (one with predators and one without). To me it just makes things more complicated.**

Response: Response: This study evaluated the effect of the number of trophic levels involved in the interactions and of the land use (not landscape) on the structure of the networks. To simplify things, we could have written a paper discussing just the interactions. However, our approach went beyond the description of the interactions. The ecology of communities changes as the number of interactions varies and as anthropogenic factors, such as land use, intervene. However, as we wrote in the Introduction, the effects of these factors do not exhibit the same trends. In addition, as Reviewer #1 stated, “The research was conducted to fill knowledge gaps in the field of ecological network analysis, particularly with regard to interactions between insect herbivores and their natural enemies and the effects of human interference on interaction networks. The authors clearly and concisely describe the current status of research on ecological networks in relation to human interference, especially invasive species. Thus, the Introduction provides ample justification for the work.” In addition, as reviewer #3 wrote, “The manuscript seeks to examine the effects of land use and community size on a plant-herbivore network system, which is a very interesting and overlooked topic in community and plant-animal interaction ecology. The importance of the study has been well contextualized and placed compared to other studies in the field.”

Consequently, we did not modify the description of the rationale of our study in the Introduction.

**L168. Please indicate which R package was used.**

Response: This has been done.

**Fig. 1 would be more useful if the species involved could be indicated.**
Response: Yes, it would be more useful. However, there is not enough space to add the names of the species to the figure. Most published papers do not include text like that because the figures get very messy. In addition, Table 1 shows which species interact with other species.

**L256-7. I don’t understand this sentence; the grammar needs to be fixed I think. I assume that the authors do not mean to say that plantations represent the ancestral state of Santa Cruz Island, but that is what the sentence seems to say**.

Response: We modified the phrase for clarity.

**L265-6. I don’t understand this sentence either. What are ‘island-like patterns’? I would think the island was more ‘island-like’ before the arriv**

Response: The phrase “Anthropogenic fragmentation of original ecosystems produce vegetation cover with island-like patterns” refers to the fact that the fragmentation of ecosystems produces patches (islands) of vegetation. This is a basic theory of fragmentation, and it is written well, in correct English. However, here is an expanded explanation: This means that the vegetation in each patch is affected by environmental factors, as occurs on real islands (see references cited afterward phrase). As stated, it refers to the effects of fragmentation in the cover of vegetation. The subject of the sentence is not the island. We believe this is clear and disagree with the reviewer’s comment.

**Reviewer #3:**

**The manuscript seeks to examine the effects of land use and community size on a plant-herbivore network system, which is a very interesting and overlooked topic in community and plant-animal interaction ecology. The importance of the study has been well contextualized and placed compared to other studies in the field. The narrative and the study design are excellent and straightforward, but the rationale needs major enhancements for more cohesion throughout the manuscript. Specifically, the research question and the objectives are not clearly stated making the story they want to highlight a little bit blurry. Additionally, tying those objectives with the methods and results needs more work as I am not sure what activities have been done to address which objectives. The authors may also want to correct the discussion accordingly.**

Response Thank you very much for your comments. We modified some parts of the manuscript for better consistency throughout the Objectives, Methods, Results and Discussion sections. We hope this is clearer now.

**L1 - Title. Could be shortened to “Land use is more of a determinant than community size of ecological network complexity”.**

Response: Thank you very much. The recommended title is shorter and more general to a larger readership. We modified it accordingly.

**L4 - Short title. Typo on “networks”; change “on” to “with”.**

Response: Thank you very much. We changed these words, as suggested.

**Abstract. The implications of your results are missing: we have the results, but what they are supposed to mean? I suggest the authors connect the results directly with their interpretation and/or implications**.

Response: We implemented this in the Conclusion paragraph.

**L23: variation of what?**

Response: Thank you very much. We changed “variation in these networks” to “variation of these networks”

**L25-27: Is the whole network comprised of exotic plant species or is there only five exotic species in the whole network system?**

Reponse: Response: The network is composed of five species of plants, which are all exotic. We changed the phrase to the following: “. … the effects of land use and number of trophic levels on the interaction networks between five exotic legume plants and the arthropods associated to the seeds of those plants. …”

**L25: are seed beetles and Bruchinae the same taxon?** If so, make sure to make that clear here as we are not all insect specialists to connect the dots between them.

Response: This was done. We modified the phrase, as shown before.

**L26: what are the four land use types?**

Response: We added the names of the four land use types

**L33-46: I suggest removing or reducing statistical reports to the minimum. I suggest focusing on the biological meaning of those statistical significances and the technical terms (connectance and IE) in the abstract to highlight your results and their implications and make it more accessible for non-network specialist scientists.**

Response: We deleted the statistical reports from the main text and added a table with this information. As a result, we think the results are easier to follow.

**L40-46: why is the last paragraph separated from the whole abstract?**

Response: This was an error. Thank you very much for pointing it out. We deleted it because it did not make sense in the abstract.

**L46. is this sentence complete or is there some explanation to it?**
Response: It was part of the mistaken paragraph that was deleted.

**Keywords. Missing**

Response: There is no keyword section in the author guidelines.

**Introduction. Excellent outline and connection of the main concepts of the studies. However, there are too many repetitions of the same information. I suggest that the authors go over the manuscript and make sure one piece of information is stated once and does not repeat it again in another paragraph. As I mentioned earlier, the research question and the objectives of this manuscript are not clear while its importance is well-outlined.**

Response: Thank you very much. We revised the entire manuscript and reviewed every section to ensure no repetition of ideas.

**L52: a consensus or evidence? Or is there any missing information?**

Response: There is consensus between those that have evidence in favor and those that have evidence on the contrary that we are in the Anthropocene. The consensus is that humans are increasingly modifying worldwide ecosystems. The sentence is correct and expresses what we meant to say.

**L54: remove abundance and richness; they are already comprised in functional and taxonomic biodiversity**.

Response: Thank you. We deleted “abundance and richness” in the sentence.
**L53-55: isn’t the loss of species that creates biodiversity functional loss? You seem to argue the opposite here**.

Response: That is not the intended meaning of that sentence. We wrote that those factors have resulted in the loss and extinction of species. The sentence is correct, and we did not change it.

**L57-58: remove “in addition to other factors”.**

Response: We deleted “in addition to other factors”.

**L62: can you advance one or two reasons why the effects of anthropogenic activities on plant-herbivore system have been disregarded despite their importance in the ecosystem**

Response: We removed this sentence from this part and added some references to explain these four lines later in the document.
**L65-66: this sentence needs a little more explanation.**

Response: We added references to support this. However, we do not believe more explanation is required.

**L67: is there a gap of knowledge on the ecology of plant-herbivore and parasitoids, or on the effects of anthropogenic activities on this system? If both are lacking, I suggest expanding more on both to highlight the gap and the importance of addressing it.**

Response: Thank you very much for your comment, in order to response it, we added some additional information (lines 55-61)

**L68-70: As this is an introductory sentence, maybe add another reference that is not focused on plant-herbivore network, such as plant-pollinator or plant-seed disperser network.**

Response: We included new references about it (line 64)
**L70-72: not clear.**

Response: We disagree with the reviewer. We did not change the sentence. Neither of the other two reviewers commented about this.

**L74-76: maybe synthetize reference 13, 17, 23-25 into one sentence or two to show the change in ecological networks in response to the introduction of exotic species instead of just citing them.**

Response: Again, we decided to leave the sentences as they are. We do not think it is necessary to explain this further.

**L77: cite both the original reference 26 and its erratum.**

Response: This has been done.

**L779-89: why do you come back to the impact of anthropogenic activities? I suggest this paragraph focusing on plant-herbivore network, what is known, and what is lacking, then introducing the lack of investigation in this field and the contradictions you found in the existing studies. Also, make sure to introduce the meaning of specific terms such as connectance. Finally, reorganize the ideas to avoid the back and forth between mutualistic networks and plant-herbivore networks.**
Response: We put this paragraph back because we cannot write about the effect of anthropogenic changes in interaction networks without describing what an ecological network is and its advantages for analyzing community structure. Thus, we left the order of the paragraphs as it was previously. However, we did add Lines 71 –73 and 81-84 to describe the relevance of studying antagonistic interactions.

**L90-91: any interaction networks? Or just plant-herbivore networks?**

Response: All of them

**L99-113: please move the information about the study site, study species in the Materials and Methods, and replace this last section with the goal and objectives of the manuscript t as well as the study system you investigate. Maybe consider also explaining why Legumes are a big deal in this ecosystem.**

Response: Thank you very much. We made the changes as suggested. Lines 124-131 explain why legumes are important to this ecosystem.

**L113-116: can you explain briefly how and add relevant references? Also, make sure to link this information about natural enemies in the rationale of your study design.**

Response: This is partially explained in the two lines that follow that statement. We included additional lines regarding the natural enemies in the rationale of the study.

**L116-120: please reorganize those sentences with the goals, objectives, study system above. Also, make sure to highlight why you specifically chose the arid zone among the other zones you mention in the study site section. Finally, I suggest you add a sentence or two to explain why you chose to examine two types of network system PSP and PSPP.**

**“Please reorganize those sentences with the goals, objectives, study system above.”**

Response: This has been done.

“**Also, make sure to highlight why you specifically chose the arid zone among the other zones you mention in the study site section**”

Response: This has been done.

“**Finally, I suggest you add a sentence or two to explain why you chose to examine two types of network system PSP and PSPP**.”

Response: This is explained in the paragraph in Lines 100 to 103. We also added Lines 94-96 at the beginning of the paragraph outlining the objectives.

**Materials and Methods. It is a very concise section although some key points are missing. However, I don’t think that is appropriate for such a technical manuscript. I suggest adding more details on some aspects of the methods, especially the data analysis (detailed below) and restructure the process following the research goal and objectives.**

**L126-127: not clear and cite only the relevant references.**

Response: We changed the sentence and cited just the most relevant reference.

**L128-131: why is this information relevant here? If it denotes the different ecosystem of sampling sites, please say so.**

Response: We changed this section entirely to make it clearer.
**L134: sampling of what?**

Response: We changed the title to “sampling and identification of organisms”.

**L135-136: I am not sure I understand whether you sampled in the vegetation zone or the arid zone, or these two are the same.**

Response: We changed the description of the area of study and the methods to make it clearer.

**L136: repetitive information.**

Response: We changed this section entirely to make it clearer. **L136-138: in how many sites did you sample exactly, five or ten? You say ten but only listed five.**

Response: We changed the entire description of sampling to make it clearer.
**L138-141: is there any particular reason to choose only these five species?**

Response to all four comments: Thank you very much. After reviewing your comments, we realized that this paragraph was not clear enough; therefore, we changed most of it. In addition, as we mentioned in the first version of the manuscript (Line 140), the seed pods were collected according to their availability. Thus, we did not choose these five species. They were collected because those species were the species that had mature seedpods during the time the field work was conducted.

**L146-147: 250 g of seeds from which species?**

Response: We changed the phrase to “we obtained approximately 250 g of seeds from each of the species found in each land use type”

**L148: please define the acronyms before using them. Also, check other acronyms throughout the text. Are these conditions standardized or is there any reason to choose them?**

Response: We revised all abbreviations. “oC” and “RH” are universal abbreviations for degrees Celsius and for relative humidity, respectively, so we did not define these.

**L153: delete the “,” between Coleoptera and Bruchinae. I suggest mentioning at the beginning of this section that you sample for those insects specifically as I am not sure if you sample for them specifically or for all arthropods earlier and then you only use identification keys for the Bruchinae here.**
Response: We eliminated Bruchinae because the references refer to Coleoptera families and species and Bruchinae, as mentioned in the lines before “The seeds were inspected every 12 hours for approximately 20 days to collect all emerging arthropods”. Then, we wrote: “Preserved arthropods were identified to the lowest taxonomic level”. We believe this is clear enough, so we did not change the paragraph in response to the reviewer’s comment.

**L161: Data analysis. Please consider reorganizing the process of analysis. There are too many “first steps, I am not sure which one goes with which one. Also, I suggest to start with the construction of the network, then the process before the actual analyses.**
Response: This was done based on the recommendations of the reviewer.

**L162-164: Networks of what? Also, how did you characterize the networks from the sampling?:**

Response: We modified the paragraph. In addition we changed “networks” for “interaction networks”
**L166-168: What kind of variation? Also, do these land use types correspond to the number of sampling sites you mention earlier?**

Response: We changed the paragraph.
**L170: Change to “This index compares samples…”**
Response: This has been done.

**L172-174: It is a bit unclear, please consider revising the sentences. Also, did you use the index for your analysis? If so, please state so. If not, please explain better why this step is critical to your data analysis.**

Response: We already wrote “Differences among the metrics of the network in response to land use and community size were calculated using a GLM at p ≤ 0.05. Sampling intensity was used as a covariate because the parameters of the networks could vary in response to the size of the sample”. However, because we changed the paragraph, we think this issue has been resolved. Please see lines 213-225.

**L178-180: Change to “This index determines the sensitivity…”**
Response: This was done.

**L190: Please explain the acronym before using it. Additionally, please make sure to mention the predictors and covariates, how many models did you run, and are there any other steps you took during the comparisons, if any.**

Response: This was done.

**L192: You can delete this sentence.**

Response: This was done.

**Results. Again, good job on the succinct writing composition! Based on the research goal and objectives here, this section fairly addresses them. I suggest revising the section by adding more structure and make the ideas better connected to fully answer the questions. Also, please do not repeat the results in the table in the text, and consider spacing the captions from the main text.**

Response: Thank you very much for your comment. We revised the manuscript and made changes and additions for more coherence.

**L196-198: are these species plant or an insect species?**

Response: As the phrase suggests, there were five species of plants with 5552 arthropods from six orders, 11 families and 17 species forming 30 interactions. To clarify, we changed the phrase to “five species of plants with 5552 arthropods belonging to six orders, 11 families and 17 species, forming 30 interactions” Table 1 was is added with the species interactions

**L198-200: Was the sampling intensity applied to both plant and insect species or plant species only? And between which variables was it compared to (sites, etc.)? Please explain these more in the data analysis section**.

Response: It includes both plant and animal species. We provided a more detailed explanation of this index in the Methods section (Lines 213-223).

**L200-202: Please write the full value of P if > 0.05. Also, correct throughout the text.
Table 1: Please check to number of sampling sites throughout the text and make sure it is always the same. Sometimes, it is ten, sometimes five, and sometimes eight. Add the caption to the table right after the title and not after the table.**

Response: We added the P values from the chi square test. We revised Table 1 accordingly.

**L211: Delete the repeated “PSPP”.**

Response: This was done. In fact, we changed the table and revised its description.

**Figure 1: Also, please add an annotation of these indications directly on the figure (L218-221).**

Response: This was done.
**Table 2: Is there any way for this table to be combined with Fig. 1? It is a bit confusing as it is now and you can color-code each insect order for instance.**

We examined the figure, and each color code represents a guild. Therefore, we cannot designate a color for each order. We changed Table 1 to make it more informative.

**L227-241: can’t the stats be added to Table 1 or another table? It becomes daunting to read. Also, please consider revising the result report. I suggest starting with the PSP community first and report all results related to it, and then the same with the PSPP.**

Response: We included a table with these results. We reported the results to compare the factors we evaluated. Thus, we did not change the sequence of the results.

**Maybe also add some basic interpretation of the comparisons for each index here as for now it looks like a repetition of what the stats show**.

Response: We changed the tables to make the results clearer and more readable. A basic interpretation of the results has been provided in the Discussion section.

**Discussion. It is a sound and thorough discussion. Similar to other section, please reorganize the ideas to reflect more the research goal and objectives and the results report. Also, it becomes a bit unclear if it is the invasive species or the land use that really threatens Santa Cruz biodiversity in the context of this manuscript. It is ok to have them both here and it is well-thought, but consider restructure the arguments so you avoid repetitions and have better implications on the negative impacts of anthropogenic activities.**

Response: Thank you very much. We modified some paragraphs in the Discussion section. Based on these comments, we changed the paragraph on *L. leucocephala* to make it more consistent with the Discussion and added some lines in the Conclusion too.
**L247-248: you did or you didn’t?**

Response: The answer is both; in some cases, we did and in others we did not. As the paragraph says, there was no difference in these parameters among the networks when comparing PSP and PSPP. However, we did find differences in the networks with respect to land use. It states the differences between the stages of the two factors evaluated: 1. number of trophic levels, and 2. among land uses.

**L248-252: please revise the connection between these two sentences.**

Response: We revised the connection between the sentences; it is explained in the following sentences at the end of the paragraph.

**L252-254: please make sure to recall your hypotheses/predictions or research questions before saying the results turned out the be as expected.**

Response: Thank you very much. We modified the sentence to clarify our meaning (see Lines 278 to 293).

**L256-264: to which results this section corresponds to? Please add this before discussing the land use and your proposed hypotheses.**

Response: We modified the paragraph because we noticed that it was not in line with the discussion.

**L272: Change “In our case” by “In addition”.**

Response: Thank you for the suggestion, but it changes the meaning. We wanted to compare what was reported previously to what we found. Thus, we believe “in our case” suits that purpose.
**L272-273: Change to “roadsides had more diverse species and interactions”.**

Response: Thank you very much. We modified the text, as suggested, for clarity.

**L273-275. This needs references, or mention it if it is a person observation.**

Response: Thank you very much. It was modified to clarify that it is a personal observation
**L280-282: This is repetitive.**

Response: No, it is not repetitive because we are showing that in our results, the connectance value of 1 does not correspond to a well-preserved ecosystem. Instead, it corresponds to the plantation that has only one species of plant. Consequently, we left the sentences as they were previously.
**L283: Ok, so what alternative do you propose?**

Response: We do not propose any alternative. That is why we wrote the following: “we cannot interpret the significance of this metric to community structure” However, we added some sentences to the paragraph to clarify our ideas regarding this. Please see Lines 320-326.

**L292: I suggest you add a paragraph talking about the conservation implications of your findings and/or the importance of addressing the plant-herbivore systems as you pointed out the lack of studies in this field and its importance for the ecosystem.**

Response: We expanded the Conclusion paragraph. (Lines 334-342).

6. PLOS authors have the option to publish the peer review history of their article ([what does this mean?](https://nam10.safelinks.protection.outlook.com/?url=https%3A%2F%2Fjournals.plos.org%2Fplosone%2Fs%2Feditorial-and-peer-review-process%23loc-peer-review-history&data=05%7C01%7Caamarillo%40javeriana.edu.co%7C8ef14515f8a046cc6d7f08dae7b95ed2%7Cdaf7990e8a3f409c9b762a5475098000%7C0%7C0%7C638077080609422994%7CUnknown%7CTWFpbGZsb3d8eyJWIjoiMC4wLjAwMDAiLCJQIjoiV2luMzIiLCJBTiI6Ik1haWwiLCJXVCI6Mn0%3D%7C3000%7C%7C%7C&sdata=8NUTqMu%2BMlSaehotlwTIs5yT%2F85BNmPhXL5yopBoQ1c%3D&reserved=0)). If published, this will include your full peer review and any attached files.


If you choose “no”, your identity will remain anonymous but your review may still be made public.


**Do you want your identity to be public for this peer review?** For information about this choice, including consent withdrawal, please see our [Privacy Policy](https://nam10.safelinks.protection.outlook.com/?url=https%3A%2F%2Fwww.plos.org%2Fprivacy-policy&data=05%7C01%7Caamarillo%40javeriana.edu.co%7C8ef14515f8a046cc6d7f08dae7b95ed2%7Cdaf7990e8a3f409c9b762a5475098000%7C0%7C0%7C638077080609422994%7CUnknown%7CTWFpbGZsb3d8eyJWIjoiMC4wLjAwMDAiLCJQIjoiV2luMzIiLCJBTiI6Ik1haWwiLCJXVCI6Mn0%3D%7C3000%7C%7C%7C&sdata=YkxWpdh%2BiDtwtCkuLNYi%2B4gbB1FOmF82GgSr8VOeYwk%3D&reserved=0).

Reviewer #1: No

Reviewer #2: No

Reviewer #3: No

While revising your submission, please upload your figure files to the Preflight Analysis and Conversion Engine (PACE) digital diagnostic tool, [https://pacev2.apexcovantage.com/](https://nam10.safelinks.protection.outlook.com/?url=https%3A%2F%2Fpacev2.apexcovantage.com%2F&data=05%7C01%7Caamarillo%40javeriana.edu.co%7C8ef14515f8a046cc6d7f08dae7b95ed2%7Cdaf7990e8a3f409c9b762a5475098000%7C0%7C0%7C638077080609422994%7CUnknown%7CTWFpbGZsb3d8eyJWIjoiMC4wLjAwMDAiLCJQIjoiV2luMzIiLCJBTiI6Ik1haWwiLCJXVCI6Mn0%3D%7C3000%7C%7C%7C&sdata=Wyt8n0fXjbKkY14pIy2wdEBrVybQqoqu7mQBYDDsppc%3D&reserved=0). PACE helps ensure that figures meet PLOS requirements. To use PACE, you must first register as a user. Registration is free. Then, login and navigate to the UPLOAD tab, where you will find detailed instructions on how to use the tool. If you encounter any issues or have any questions when using PACE, please email PLOS at [figures@plos.org](mailto:figures@plos.org). Please note that Supporting Information files do not need this step.

General responses regarding the comments made by Reviewer #2:

We are sorry that the reviewer felt frustrated reading the manuscript. The authors also sometimes find science to be frustrating. Especially when we found reviewers that prefer to comment on a rude and aggressive way, and would prefer to comment on how a study should be done in different ways instead of giving comments in a positive way to improve the manuscript. It is challenging to address this type of comments; we would prefer comments that suggest positive ways to improve the manuscript. For example. when writing: “**I found these to be frustrating though since there was no attempt to correct these effects for host plant species.”** We interpret this comments as: “It is not clear how the metrics used accounted for the effects of host plant species, which is very important since the same species of plants were not found on each land use area”. In fact, we did correct for the effects the reviewer mentioned, but maybe it was unclear which metric we used for that. Our response to this comment and the description of the changes to the manuscript have been provided above.

**When writing: “It is not hard to imagine that the trends being ascribed to ‘land-use’ had alot (or at least something) to do with the host-plants present at these sites”** we interpreted the comment as: “please provide evidence that the metrics used to describe the networks correct for the differential composition of plant species”. Our response to that comment has already been provided above.
