# Supplementary material for: Land use is a stronger determinant of ecological network complexity than the number of trophic levels
Source: PLoS One. 2024 Feb 9;19(2):e0295377. doi: 10.1371/journal.pone.0295377 (PMC10857743; doi:10.1371/journal.pone.0295377)
Supplement: Attachment — Submitted filename: Response to Reviewers No-2 to plosOne.docx [file pone.0295377.s002.docx]

Dear Editor,

Thank you very much for all comments we obtained from the reviewers. Following you will find our response to each comment. The reviewer comment is in bold letter and our response follow the comment in normal lettering.

**Please include the following items when submitting your revised manuscript:**

- **A rebuttal letter that responds to each point raised by the academic editor and reviewer(s). You should upload this letter as a separate file labeled 'Response to Reviewers'.**
- **A marked-up copy of your manuscript that highlights changes made to the original version. You should upload this as a separate file labeled 'Revised Manuscript with Track Changes'.**
- **An unmarked version of your revised paper without tracked changes. You should upload this as a separate file labeled 'Manuscript'.**

**If you would like to make changes to your financial disclosure, please include your updated statement in your cover letter. Guidelines for resubmitting your figure files are available below the reviewer comments at the end of this letter.

If applicable, we recommend that you deposit your laboratory protocols in protocols.io to enhance the reproducibility of your results. Protocols.io assigns your protocol its own identifier (DOI) so that it can be cited independently in the future. For instructions see:** [**https://journals.plos.org/plosone/s/submission-guidelines#loc-laboratory-protocols**](https://nam10.safelinks.protection.outlook.com/?url=https%3A%2F%2Fjournals.plos.org%2Fplosone%2Fs%2Fsubmission-guidelines%23loc-laboratory-protocols&data=05%7C01%7Caamarillo%40javeriana.edu.co%7Ccd7ff305c0db40f994c208db873a9e2c%7Cdaf7990e8a3f409c9b762a5475098000%7C0%7C0%7C638252458087915807%7CUnknown%7CTWFpbGZsb3d8eyJWIjoiMC4wLjAwMDAiLCJQIjoiV2luMzIiLCJBTiI6Ik1haWwiLCJXVCI6Mn0%3D%7C3000%7C%7C%7C&sdata=IfozGR9OCmH8B9YN6KZYgw5F%2BQSfOblRfs59gruDiOo%3D&reserved=0)**. Additionally, PLOS ONE offers an option for publishing peer-reviewed Lab Protocol articles, which describe protocols hosted on protocols.io. Read more information on sharing protocols at** [**https://plos.org/protocols?utm_medium=editorial-email&utm_source=authorletters&utm_campaign=protocols**](https://nam10.safelinks.protection.outlook.com/?url=https%3A%2F%2Fplos.org%2Fprotocols%3Futm_medium%3Deditorial-email%26utm_source%3Dauthorletters%26utm_campaign%3Dprotocols&data=05%7C01%7Caamarillo%40javeriana.edu.co%7Ccd7ff305c0db40f994c208db873a9e2c%7Cdaf7990e8a3f409c9b762a5475098000%7C0%7C0%7C638252458087915807%7CUnknown%7CTWFpbGZsb3d8eyJWIjoiMC4wLjAwMDAiLCJQIjoiV2luMzIiLCJBTiI6Ik1haWwiLCJXVCI6Mn0%3D%7C3000%7C%7C%7C&sdata=VlRbTl0fRctDQHmhyEdSawVSOSYmXO4iZNg%2BmLLJKM4%3D&reserved=0)**.

We look forward to receiving your revised manuscript.**

**Kind regards,**

**Sean Michael Prager, Ph.D.
Academic Editor
PLOS ONE**

**Journal Requirements:

Please review your reference list to ensure that it is complete and correct. If you have cited papers that have been retracted, please include the rationale for doing so in the manuscript text, or remove these references and replace them with relevant current references. Any changes to the reference list should be mentioned in the rebuttal letter that accompanies your revised manuscript. If you need to cite a retracted article, indicate the article’s retracted status in the References list and also include a citation and full reference for the retraction notice.

[Note: HTML markup is below. Please do not edit.]

Reviewers' comments:

Reviewer's Responses to Questions**

**Comments to the Author

1. If the authors have adequately addressed your comments raised in a previous round of review and you feel that this manuscript is now acceptable for publication, you may indicate that here to bypass the “Comments to the Author” section, enter your conflict of interest statement in the “Confidential to Editor” section, and submit your "Accept" recommendation.**

**Reviewer #1: (No Response)**

**Reviewer #3: (No Response)**

**2. Is the manuscript technically sound, and do the data support the conclusions?
The manuscript must describe a technically sound piece of scientific research with data that supports the conclusions. Experiments must have been conducted rigorously, with appropriate controls, replication, and sample sizes. The conclusions must be drawn appropriately based on the data presented.**

**Reviewer #1: Yes**

**Reviewer #3: Partly**

Response: Based on the comments made by reviewer # 3, we believe that “Partly” refers to the conclusion section because he/she answers Yes to everything else.

**3. Has the statistical analysis been performed appropriately and rigorously?**

**Reviewer #1: Yes**

**Reviewer #3: Yes**

**4. Have the authors made all data underlying the findings in their manuscript fully available?

The** [**PLOS Data policy**](https://nam10.safelinks.protection.outlook.com/?url=http%3A%2F%2Fwww.plosone.org%2Fstatic%2Fpolicies.action%23sharing&data=05%7C01%7Caamarillo%40javeriana.edu.co%7Ccd7ff305c0db40f994c208db873a9e2c%7Cdaf7990e8a3f409c9b762a5475098000%7C0%7C0%7C638252458087915807%7CUnknown%7CTWFpbGZsb3d8eyJWIjoiMC4wLjAwMDAiLCJQIjoiV2luMzIiLCJBTiI6Ik1haWwiLCJXVCI6Mn0%3D%7C3000%7C%7C%7C&sdata=E7dyXUlbfNZoE3%2BsmDmCo%2BCc%2BReSm4PklOGzdVgCcX8%3D&reserved=0) **requires authors to make all data underlying the findings described in their manuscript fully available without restriction, with rare exception (please refer to the Data Availability Statement in the manuscript PDF file). The data should be provided as part of the manuscript or its supporting information, or deposited to a public repository. For example, in addition to summary statistics, the data points behind means, medians and variance measures should be available. If there are restrictions on publicly sharing data—e.g. participant privacy or use of data from a third party—those must be specified.**

**Reviewer #1: Yes**

**Reviewer #3: Yes**

**5. Is the manuscript presented in an intelligible fashion and written in standard English?

PLOS ONE does not copyedit accepted manuscripts, so the language in submitted articles must be clear, correct, and unambiguous. Any typographical or grammatical errors should be corrected at revision, so please note any specific errors here.**

**Reviewer #1: Yes**

**Reviewer #3: Yes**

**6. Review Comments to the Author

Please use the space provided to explain your answers to the questions above. You may also include additional comments for the author, including concerns about dual publication, research ethics, or publication ethics. (Please upload your review as an attachment if it exceeds 20,000 characters)**

**Reviewer #1: The authors have made significant changes to the manuscript (now retitled): “Land use is mor of a determinant than number of trophic levels of economic complexity.” In particular, more information is now provided in the main text and in the figures and tables to help communicate the intricacies of the experimental design. Overall, the readability of the manuscript has increased, as has the clarity. One additional comment for the authors to consider in addressing an important aspect of the methodology (sampling period) is below (pertaining to the text in L180-187). Otherwise, I suggest only a few additional minor revisions, as listed below. Thank you.**

Thank you very much for your comments. They contributed greatly to improving the quality and clarity of the manuscript. Concerning the comment on the inclusion of the sampling period in the text (L180-187), we chose not to make this change for two reasons. First, we have already addressed the sampling period at the beginning of the Materials and methods section. Second, lines 176-185 specifically refer to the permits obtained for collecting the samples.

**L64-66. Please review this sentence as there appear to be some errors and as a result, the idea being communicated is not entirely clear.**

Response: Thank you very much. Yes!! You are right. We missed some words in the new version. It is now: “Understanding how the structure of these relationships functions, changes, and is constituted, as well as how it is affected by anthropogenic activities, is necessary for understanding the cascading impact on ecosystem integrity.” **L171. Does ‘gr’ following 218 refer to grams? If so, the SI unit for grams is ‘g’**

Response: Thank you. We changed it to “g”. **L180-187. In the response to the reviewers, the authors indicated that they only sampled for one year due in large part to the need for the permits listed here. The manuscript would benefit from a statement along those same lines in the Methods, as at least two reviewers expressed concerns related to the sampling period. For example, the authors could write (starting on L187) that: “Sampling for this research was restricted to one season (August-September, 2018) due to the logistics involved with procuring permits to sample and export samples from the island of Santa Cruz.”**

Response: Thank you very much. We added a minor change as suggested. The text now reads: “Sampling for this research was restricted to August-September 2018 due to the logistics involved with procuring permits to sample and export samples from the island of Santa Cruz.” **L199. There is a typo at the start of this sentence, where “This index” is repeated.**

Response: Thank you. We deleted the repeated words. **L331. Please revise to “… and this may imply that…”**

Response: thank you. It read awkward. We changed to: Thank you. The passage read awkward. We changed it to: “However, one possible way to explain this metric is through a study that demonstrated that networks containing invasive species are more resilient to extinction events [83], suggesting that eradication of the invasive…”

**Reviewer #3: By examining an overlooked plant-arthropod network, this manuscript draws attention to the importance of antagonistic interactions in ecosystems as well as the careful steps we should take regarding eradication of invasive species. The authors have substantially improved the content of the manuscript and bring more details to their work. However, the clear rationale of the study design and objectives are still unclear, which tremendously weaken the importance of their result and the implications of such results to the biodiversity of Santa Cruz Island. I recommend the authors to thoroughly restructure the Introduction, reorganize the Methods section, and subset Results and Discussion sections accordingly for a clearer workflow and a more impactful contribution not only to plant-animal and biological invasion ecology. Additionally, I strongly suggest the authors to 1) use shorter sentences to make clearer statements, 2) detail workflow chronologically to avoid repetitions of information, especially in the Methods section**

Response: We do not agree with this comment from the reviewer. The methods are presented in a sequential manner, both chronologically and by moving from general to specific activities and procedures. This sequence is organized into three sections: first, we characterize the 'Study site'; second, we describe the 'Sampling and identification of plants and arthropods'; and third, we outline the 'Data analysis'."

Within the 'Sampling and identification of plants and arthropods ' section, we follow a sequence of steps: first, we describe when we performed the sampling; second, we explain how we selected the sampling areas; third, we outline the process of selecting the trees for seed collection within each area; fourth, we detail the seedpod collection process, followed by seed collection and data gathering from each sampled tree; fifth, we describe how we transported the collected seeds to the laboratory, monitored them, and identified the emerging insects. Finally, we conclude this section with information regarding the permits required to conduct the complete study.

Within the 'Data analysis' section, we follow a specific sequence: first, we describe the construction of the interaction networks; second, we define and explain the parameters used in the networks and how we validated the observed values obtained. Finally, we explain how we compared the metrics of the networks.

**and 3) thoroughly clean and proofread the final version to make sure all components of the manuscript are coherent, complete, non-repetitive, and easier to read. I provide more detailed recommendations below.**

Response: Thank you very much for the comment. We revised the entire manuscript to improve clarity and coherence.

**Abstract. It has been improved but needs more cohesion between the different sections. Please refer to the guidelines for the information to include for the abstract. Please group information per section as well: background, objectives and hypotheses, methods and analyses, results, and implications, etc. There is currently too many back-and-forth information in this abstract, which leads to confusion. I also suggest removing acronyms as much as possible.**

Response: We revised the abstract in accordance with the journal's guidelines, which do not require grouping information by sections. As per the guidelines, the abstract should describe the main objectives of the study, provide an overview of the study's methods without going into methodological detail, summarize the key results and their significance, and not exceed 300 words. However, we made sure to incorporate the elements mentioned by the reviewer where they were relevant. We did not include a hypothesis since we did not have one prior to the study, and it would not be accurate to introduce one after the study was completed. Additionally, we adjusted the abstract to adhere to the 300-word limit.

**L17-18: Are you looking at the impact of land use of invasive species? I understand both are somewhat related, but you need to highlight what aspects of such relationship or which of them pertains more to your work. Perhaps defining that scheme may help structuring your line of ideas and maintain them throughout the abstract and revise the manuscript accordingly.**
Response: Thank you for the comment. It has us working in the better way to show our study. Lines 17-18 are the first two lines of the abstract. They do no refer to the question the reviewer wrote. However, as the 22-24 stated: “we analyzed the effects of land use and number of trophic levels.

We did revise the abstract, and changed a part of it to make it more coherent with the entire paper. When revising the coherence of the paper we found this: The objective literally says in the abstract: “We analyzed the effects of land use **and** number of trophic levels on the interaction networks between exotic legume species and their associated arthropods.”

Then, in the introduction says: “Studies analyzing how the parameters and structure of networks change when considering simultaneously the number of trophic levels and a variety of Thank you for the comment, it helped us improve our manuscript. Lines 17-18 are the first two lines of the abstract. They do not refer to the question the reviewer asked. However, as lines 22-24 stated, “we analyzed the effects of land use and number of trophic levels.”

We revised the Abstract and changed a part of it to make it more coherent with the entire manuscript. When revising the coherence of the text, we note that the objective is clearly stated in the Abstract: “We analyzed the effects of land use and number of trophic levels on the interaction networks of exotic legume species and their associated arthropods.”

Then, the Introduction states: “Studies analyzing how network parameters and structure change when simultaneously considering the number of trophic levels and a variety of land uses are important for testing the multifactorial causes determining the responses of biodiversity to environmental change. Thus, the objective of this study was to

analyze the effect of land use and number of trophic levels

**on**

the structure and complexity of the interaction networks

**between**

arthropods and plants associated with the seeds of five species of exotic leguminous trees…”

This is followed by:

“To do this, we defined two communities that differ in the number of trophic levels on four land uses.”

We changed some phrases, especially the order, of some parts of the Methods section to align it with the introduction, objectives and methods: we defined what four land uses we identified and described them, and in the analysis section, we presented the two communities we compared: three trophic levels vs. more than three trophic levels.

In the Results section, there is a paragraph that already reads: “Hereafter, the results are described for each metric regarding the two factors evaluated: number of trophic levels and land use.” Thus, we described the effects in that regard.

In the Discussion section, we start by writing: “Our study analyzed the effect of land use and number of trophic levels on the structure and complexity of the interaction networks of insects and plants associated with the seeds of five species of exotic leguminous trees…”

Finally, the concluding paragraph states: “In conclusion, the most relevant factor responsible for the differences in the structure of arthropod communities associated with the investigated five legume species was land use type rather than the number of trophic levels involved in the analysis.”

Thus, we are confident that the modifications we made show a clearer progression of ideas, which are maintained from the Abstract to the Conclusion.

**L18-22: That is a very long sentence, and it does not help stating the knowledge gap and study objectives. Please reformulate.**

Response: We split the sentence into two parts, aligning it with the modifications we made to the abstract.

**L23 & 28: Please use consistent terminology arthropods or Bruchinae throughout the abstract and manuscript as it can be misleading, especially for readers who are not entomologists.**

Response: Thank you for your comment. We have revised the entire manuscript accordingly.

**L25: I don’t think you need to detail this here.**
Response: Thank you for the comment. We decided to leave this sentence as it was, because it is part of the methods.

**L27-30: Please explain the rationale between the use of two different community compositions. Perhaps, removing “three levels” and “more than three levels” and shortening the sentence can help reducing the confusion.**

Response: It is a part of our aims: to determine differences in the networks due to communities of different sizes as it is explained in the sentence: “We analyzed the effects of land use and number of trophic levels on the interaction networks of exotic legume species and their associated arthropods” in the Abstract. We also explain this in the Introduction.

**L30-38: Please present concisely the most relevant results related to your objectives here and move the analysis-related information into the appropriate section.**

Response: We decided to leave the results as they were. It is clear that we stated the main results in this part of the abstract.

**L38-42: This is another long sentence, please split it and separate the implication of your work and the potential impacts of your work.**

Response: As we adjusted the abstract to the number of words required, this sentence was also changed. **Introduction. The study rationale still looks blurry to me, which preclude the understanding of the overall importance of this work. The different paragraphs were very much improved but needs a bit more cohesion and details to make sense of the reasoning behind each argument and highlight the actual contribution of the manuscript.**

Response: This comment and some others made by the reviewer contradict the evaluation given by the reviewer in question #5: Is the manuscript presented in an intelligible fashion and written in standard English?

The answer was yes. This means that the manuscript was presented in an intelligible fashion. Reviewer #1 answered the same way. Thus, the reviewer answered yes to question #5, but in the comments and regarding the methods the reviewer suggests that the rational is blurry. **L49: What is the evidence? Please expand a bit your argument here or provide specific examples.**

Response: This is explained already in lines 50-52 in the original version. Thus, we decided to leave the paragraph as it is.

**L55-56: What three factors? And how do they modify the structure? Please expand.**

Response: The three factors are mentioned at the beginning of the sentence: Fragmentation, habitat loss and exotic species introductions. This comment has the same problem as any other comments we have already answered: it is based only on the sentence without considering the sequence of ideas in the paragraph. In addition, the Introduction comprises 3.25 pages, which is an average length for this type of study. If we explain and expand each sentence, it would be much longer and confusing. The study is directed toward an expert audience. Thus, we believe that too much explanation and expansion of each sentence would be detrimental to the coherence of the text.

**L56-57: If you want to contrast the attention received by mutualistic and antagonistic interactions, I suggest start a new paragraph with this sentence and restructure the Introduction accordingly. This is also a great place to highlight how important antagonistic interactions are in different ecosystems.**

Response: Thank you for the first part of your comment regarding the comparison with mutualistic interactions. After considering it, we decided to delete that phrase and lines 61-64 of the original version. It did not aid in the development of the ideas. Regarding the mention to highlight how important antagonistic interactions are, that is what we touch on in the following lines of that paragraph.

**L61-64: These sentences need a better transition, and please revise it if you consider my suggestion above.**

Response: Thank you for your comment. We considered it and deleted the text accordingly.

**L73-76: Please consider spreading the references to each of the arguments you are using instead of having a long list at the end of the sentence.**

Response: Done.

**L76-77: Isn’t this sentence a consequence of anthropogenic perturbations as well? Why is it separated? Please consider a better transition.**

Response: It is separated from the preceding sentence because it explains that the changes could also sometimes, but not always, lead to extinction of populations and species.

That is why we write “Such perturbations can even push…”.

**L81: What parameters are you considering here?**

Response: The parameters of ecological networks. This is the name given to estimators such as connectance, nestedness, etc. And it is explained in the following lines of the paragraph.

**L85: “While there was no border effect for such parameters”? Please clarify.**

Response: Again, this is article is intended for an expert public. The example explains the effect on the network parameters of communities exposed to habitat fragmentation.

**L87-90: This is a very long sentence and has repetitive information. Please reformulate.**

Response: Thank you. The sentence is no longer repetitive and long since we deleted the text referring to mutualistic interactions. We also deleted the reference to mutualistic interactions in these lines.

**L93-99: Is there any reason these details are presented in the Introduction? I suggest removing them or putting the essential information in the Methods section. Also, please remove repetitive information.**

Response: The reason is that it shows that exotic species and human activities are a major threat to the specific place we are studying: the Galapagos. The entire Introduction states the general effects of such perturbations on biodiversity, and this paragraph shows the context for the area we are studying. If we remove the paragraph, the Introduction would be too general and would say nothing about the challenges the Galapagos Archipelago faces because of those activities. The beginning of the sentence states: “The Galapagos Islands are an ideal model for analyzing the effects of anthropogenic perturbations on interaction networks.” This is not just because the islands are highly diverse but also because they have been subjected to challenging conservation issues because of the main human activities carried out there. Given these reasons, we decided to leave these sentences in the Introduction.

**L103-106: This is a very long statement of objective. I suggest presenting the objective more concisely and then in another sentence or two present the system you work with to reach this objective.**

Response: We changed the sentence to make the objective clearer.

**L106-110: I don’t think this information is necessary in the Introduction. I suggest presenting hypotheses and/or predictions here along with the rationale behind why you want to look at the effects of trophic levels instead. You may also want to add a sentence or two stating the knowledge contribution of this manuscript or a long-term goal of such study.**

**Response:** We did not include a hypothesis since we did not have one prior to the study, and it would not be accurate to introduce one after the study was completed **Methods. There are a lot of repetitive information throughout this section. Additionally, I still can’t make sense of the importance of the activities towards the study objective. As I said in my previous review, please consider stratifying this section. You can do so by 1) briefly introducing the general approach you use to reach your objective, 2) giving separate title to each of the activity so readers can follow which activity pertains to which part of the objective, and 3) using chronological order to describe your activities.**

Response: We started the Methods section by describing the study site; then, in a different subsection, we describe how and where we collected the samples. In a separate paragraph, we describe how we obtained the arthropods, how we preserved them, and later how we identified them. After the subsection on sampling and identification of organisms, there is a a third subsection titled “Data analysis”. In this subsection, we write separate paragraphs describing the type of networks we built, then another paragraph that explains the metrics we estimated for the networks, and a last paragraph describing how we compared the networks. We strongly consider that the methods are described in a sequential and rational way, as recommended by the reviewer. You cannot analyze data without identifying the species, and identification of species cannot be done without sampling the arthropods. Similarly, in the data analysis, there is no possibility to compare metrics among the networks if there is no estimation of the metrics first. This comment by the reviewer does not make sense after she/he answered that yes, the rationale of the manuscript is presented in an intelligible fashion. The methods were changed in some parts after considering the comments of both reviewers.

**L117-120: I don’t think this piece of information is necessary since you already describe the climate of the “actual” study site further below.**

Response: We consider that the reviewer is mistaken regarding the descriptions. The first one refers to the mean temperatures of the rainy and dry seasonal periods. The other reference to temperature refers to the mean temperature of the arid zone. We thus left this paragraph as it is. **L121: Maybe start a new paragraph with the description of Santa Cruz Island.**

Response: Done.

**L124-127: These sentences need a better transition with the previous and following information.**

Response: We changed the order of sentences and paragraphs and wrote new sentences in this sections. We believe it is clearer now. **L127-130: Why coming back to the Galapagos here? Why don't you focus only on Santa Cruz Island?**

Response: Because we changed the subject and started writing about legumes in the arid zone. **L130-134: I suggest moving this information in the Introduction when presenting the system you are working with?**

Response: You are correct. We moved this information to the Introduction.

**L135-141: Please combine this information with the description of Santa Cruz Island earlier and remove repetitive information. Also, consider explaining why you chose Puerto Ayora among many other sites in Santa Cruz Island.**

Response: We changed the sentences and rewrote this part, indicating the reasons to conduct the study in Puerto Ayora.

**The description of the four land use types is also missing from this section, or if there is any, please put them after or with the description of the study site.**

Response: We moved this information from the Sampling section to the Study site section as recommended. **L143: This subtitle does not tell much. What “organisms” are you referring to? Please use more informative or descriptive subtitle, perhaps you can put “Seed collection”.**
Response. We changed the subtitle to “Sampling and identification of plants and arthropods”.

**L144-145: Are there any specific species you collect the seeds from? If so, what are these species and why did you focus only on them?**

Response: This information is presented at the end of the same paragraph containing these lines.

**L146-150: I am not sure about the relevance of these sentences here. Please clarify or restructure.**

Response: We do not understand the rationale of the reviewer’s comment. In some notes above, the reviewer asks for a description and an explanation of why we conducted sampling in the arid zone; the explanation here is about that. Then the reviewer comments that this is not relevant. We consider the sentence to be relevant and decided to leave it as is.

**L152-154: Did you choose the trees before the land use types or vice-versa? This needs a clarification as your whole study lays on the impact of land use and if you chose the trees before the land use, it introduces a bias in your study design.**
Response: The methods are written in the order we performed the sampling. We sampled trees that were found on all the land uses that existed in the sampling area and those that had mature seedpods. Thus, there is no bias because there were no more land uses in the sampling areas with legume trees and with trees with mature seedpods.

**L154-162: This is too confusing, there is too much information to take in all at once. Perhaps a table might help: you can add the land use type with its description and the tree species you sampled there with the number seeds collected. Also, if you want to highlight the importance of Leucaena leucocephala or any other exotic and invasive species, you may want to do so in the description of your study species, which you can put right after the description of the study site.**

Response: The table described was included in the first version of the manuscript, it is Table 1. We added this information because it was required by reviewer #3 in the first revision of the manuscript

**L168: This paragraph and the next could be under another subtitle. You may put “Collection and identification of Arthropods in the seeds” or something along that line.**

Response: This paragraph follows the paragraph before on collecting the seeds in the field. We believe that if we put this information in another section we would have to define a section for field collection, another for transport to the laboratory, another for identification of organisms (plants and arthropods), etc., and there would be too many subsections. We decided to leave this part as is: a different paragraph for each step of the “Sampling and identification of plants and arthropods”.

**L180: Please separate this paragraph from the previous one.**

Response: Done **L188: You may also want to chronologically stratify the different steps of your analyses here as there is a lot of back-and-forth in the process**.

Response: Once again, we disagree with the reviewer. We started this section with a paragraph describing the general steps. Then, we dedicated the second paragraph to explaining how we built the networks, followed by another paragraph indicating what parameters we estimated for the networks, and last, a paragraph on how we compared them. We decided to leave this section as is. In addition, reviewer #1 agrees with us and made no comments on this subject.

**L190-192: Please consider explaining the differences between interaction network and trophic network. Those terms are sometimes used interchangeably in the manuscript and in other times as different entities.**

Response: We revised the manuscript and corrected it as recommended. **L193-197: You need to be more specific here. Did you compare the network across the land uses or between the different trophic levels? Or you compared both at the same time? I suggest describing the models you fitted directly here with the independent and dependent variables you used in your analyses.**

Response: We changed some lines in the paragraphs in this section to make it clearer. In addition, a detailed explanation is given in the last paragraph of the section.
**L198: You may to present why you are looking at these parameters regarding your objective. Then you may want to present these parameters and their description in a table or a more concise way. They are a bit confusing presented like this.**

Response: We decided to leave the description of the metrics used in the analysis as is, in paragraph form. These are just tree metrics. We consider that this would be a very small table and would not add more clarity; we would just be writing the description in a table instead of presenting it in a paragraph. Instead, we added one concise sentence at the beginning of the paragraph and explained why those parameters were estimated.

**L212: You can include this sentence with the previous two and perhaps in a separate paragraph.**

Response: Done

**L213-225: How different from L208-211 is this section? I am confused. Perhaps this suggestion can help: you can describe the network first and present any statistical analyses you did along with it, and then compare the network between/across land uses and trophic levels with the statistics.**

Response: These greatly differ. L208-2011 explains how we validated the parameters we calculated for the networks if they were significantly different from a null model, and L213-225 explains how we compared the metrics between land uses and number of trophic levels. We cannot present the statistical analyses we performed. We explained them. This is the methods section.

**L221: What does a “GLM at p < 0.05” mean? Please also consider adding the independent and dependent variables of your models.**

Response: We changed the test o “at a significance level of p ≤ 0.05”

**L224: What is a “null model t model analysis”?**

Response: We changed to: “…, we performed a comparison with null models using the t test” **Results. There is much improvement in the presentation of the results. However, the actual link with the objective and the Methods section is still blurry. I suggest presenting the results in accordance with the different subsets of the Methods, which can also help avoiding repetitive information. You may also want to use the same chronological order as in the Methods here to make your results more impactful and clearer. Please do not repeat the figures and tables in the text, but rather provide a little interpretation of the results. Finally, when presenting statistics in the results, please remind us the test you used as you used at least two tests.**

Response: We changed some parts of each section of the manuscript to make it clearer. Most of the statistics presented are summarized in Table 2. The only statistics presented in the text are those testing the effect of sampling intensity among land use types at any number of trophic levels; in that case the manuscript states “X^2”^, which universally means Chi square. Thus, we did not change the text.

**L229-231: Please double check the number of arthropods and seeds you collected. There are some contradictions here and in the Methods.**

Response: We double checked the numbers in the Methods and Results, and we did not find contradictions. In the Methods section, we refer to the average grams of seeds obtained from each species, from each land use and to the average number of seeds per species. In the Results section, we report the total number of arthropods and the total number of seeds collected.

**L231-233: Are these legume or arthropod species? A subtitle of the results may help in indicating the reader what species you are talking about here.**

Response: We changed to “the most abundant species were the beetles.”

**L235: Perhaps this could be another subtitle or a new paragraph, depending on the restructuration you bring to the Methods.**
Response: Following the order we stated in the Methods, we separated this part to compose a new paragraph.

**L237-241: Perhaps you could combine these two sentences and highlight directly which of the results did not have significant differences instead of separating them, it’s a bit confusing.**

Response: We changed text and combined the sentences as suggested.

**Table 1. Associations between plants or seeds? You may want to be clear about that because it can lead to a confusion. You can delete “Species of arthropods … Island of Santa Cruz” as it is redundant to the table title.
Table 1.**

Response: It is an association between arthropods and plants because we are reporting the species of plants to which the seeds belong. We deleted “Species of arthropods … Island of Santa Cruz”

**Table 2. Please move the title before the table. Also, there are two exact same table 2 in this text, please make sure you only have one. You may also want to merge the cells indicating the same land use types as having two consecutive rows with the same contents are confusing. What p-values are you presenting here? What are you comparing here? Remind us of the statistical test you used in the comparisons.**

**Table 2.**

Response: We moved the title before Table 2 and eliminated one of the repeated tables. We also eliminated the repeated names of the land uses in the first column. Information regarding the statistical test used and the meaning of the p values was added to the description of the table.

**Table 3. Which covariates and metrics are you examining here? To which models are you referring to? Remind us of the statistical test you used and please define F, P, and DF as they are not informative at all. You may also add a bit explanation of this table in the tex.**

Response: The reviewer is correct. Table 3 confuses and is not informative. We eliminated this table.

**Figure 1. Are the parasitoids and phytophagus associated with the arthropods or the plants or the seeds? You may want to explain that when you present your choice to examine the effects of trophic levels. Also, what is LSPPP and LSP? Are they related to PSP and PSPP or different systems? Please explain.**

Response: At this point, we apologize for not complying with many of the comments made. However, we strongly believe it is clear that when we mean the “number of trophic levels involved in the analysis”, we mean that in some analyses, we included only “plant–seed feeders–parasitoids” (three levels); in others, we included more than three levels or “PSPP”. This is explained throughout the entire manuscript. We changed some parts of the description of the figure to make this clearer.

**L270-282: I suggest you remain consistent with the structure of your results. Either you describe the results and provide a concise interpretation for all of them, or you only present them.**
Response: We mostly report results in the section you mention. To be consistent, we eliminated the phrase “the networks were functionally redundant”.

**L277: What does “the networks were functionally redundant” mean? What aspects of your results allow you to state so? Please explain, and I suggest putting such speculation in the Discussion.**

Response: We eliminated this sentence after considering that it will not improve the Discussion.

**L279-280: This sentence does not inform much about your results; either it misses some interpretation, or it needs a better transition with the previous and following statements.**

Response: This sentence goes along with the paragraph because in the preceding lines, we are reporting the results of each network parameter, and in these lines, we report the results for ISA. The lines do not stand alone. They mention that there was a negative ISA value for a land use and a given number of trophic levels, and the next paragraph explains what the values were for the remaining one. Consequently, we decided to leave the sentence as is. **Discussion. As you revise the structure of your Methods and Results section, please also consider this section to follow the different subsets you are using.**

**L285: Please stay consistent with the wording: arthropods, insects, or Bruchinae.**

Response: Revised and adjusted.

**L288: Did you already explain what you mean by community size in the Introduction or Methods Section? If not, please do so.**

Response: We explained this in the same line to make a connection with our study.

**L293: “Preserved and continuous forests”**

Response: Changed as suggested.

**L296-299: This interpretation (or implication?) needs a better formulation. What did you consider, and what does that imply?**

Response: The sentence is clear: when only number of trophic levels were considered, and after, the text states “the results were similar to those found in other studies; **that is**, the number of trophic levels directly affected the diversity of the interactions. That is = meaning that.

**L301-302: How relevant is this sentence here? Please revise the transition with the other information.**

Response: We revised this paragraph and changed to “One of the more conspicuous plant species in the network deserves special mention: the invasive *L. leucocephala*.”

**L307: Preservation of what?**

Response: The phrase should be read complete and not in a fragmented way. We wrote “Because it is an invasive species, it could also negatively affect local populations of native legumes [38] and even become a threat to their preservation on the island.” Thus, we refer to the preservation of the subject in the phrase “populations of native legumes”.

**L309: Which hypotheses are you referring to?**

Response: We changed the word “hypothesis” to “assumptions”.

**L311-313: Why are you comparing seed dispersal interaction to an antagonistic interaction? I don’t think this is relevant here as the two interactions have completely different dynamics and structure, thus your comparisons might not be valid even though both are impacted by land use types.**

Response. We found the argument valid. We deleted this comparison.

**L317-318: What species and interactions?**

Response: The species and interactions we are analyzing in the study.

**L320-321: This closing argument contradicts your whole paragraph. Is this intentional or is there any information missing?**

Response: We deleted these sentences.

**L322: Better briefly describe the system of this study.**

Response: We deleted the sentence because the comparison was out of place. **L327-328: High value of what?**

Response: We changed this term to “high connectance value”. **L330-333: Please consider a better transition with your results here.**

Response: We changed the transition connector in the phrase**.
L340-343: I am not sure about the relevance of this statement here. Please consider a better transition.**

Response: We changed the phrase to make it clearer. **L345: Start of what actions?**

Response: We eliminated these words. To explain them would be redundant as the paragraph stars by stating “The development of plans for the eradication or control”. Thus, those are the actions we refer to.

**7. PLOS authors have the option to publish the peer review history of their article (**[**what does this mean?**](https://nam10.safelinks.protection.outlook.com/?url=https%3A%2F%2Fjournals.plos.org%2Fplosone%2Fs%2Feditorial-and-peer-review-process%23loc-peer-review-history&data=05%7C01%7Caamarillo%40javeriana.edu.co%7Ccd7ff305c0db40f994c208db873a9e2c%7Cdaf7990e8a3f409c9b762a5475098000%7C0%7C0%7C638252458087915807%7CUnknown%7CTWFpbGZsb3d8eyJWIjoiMC4wLjAwMDAiLCJQIjoiV2luMzIiLCJBTiI6Ik1haWwiLCJXVCI6Mn0%3D%7C3000%7C%7C%7C&sdata=0htuPVAEGuw%2B2JoQAxa0bJt3Sz0P4ver8AyO0GkId0I%3D&reserved=0)**). If published, this will include your full peer review and any attached files.


If you choose “no”, your identity will remain anonymous but your review may still be made public.


Do you want your identity to be public for this peer review? For information about this choice, including consent withdrawal, please see our** [**Privacy Policy**](https://nam10.safelinks.protection.outlook.com/?url=https%3A%2F%2Fwww.plos.org%2Fprivacy-policy&data=05%7C01%7Caamarillo%40javeriana.edu.co%7Ccd7ff305c0db40f994c208db873a9e2c%7Cdaf7990e8a3f409c9b762a5475098000%7C0%7C0%7C638252458087915807%7CUnknown%7CTWFpbGZsb3d8eyJWIjoiMC4wLjAwMDAiLCJQIjoiV2luMzIiLCJBTiI6Ik1haWwiLCJXVCI6Mn0%3D%7C3000%7C%7C%7C&sdata=1zPnPDmP6LDRgbgBvIwDe8VAvfuP4cZ1%2FPmKwYZ14pc%3D&reserved=0)**.**

**Reviewer #1: No**

**Reviewer #3: No**

**[NOTE: If reviewer comments were submitted as an attachment file, they will be attached to this email and accessible via the submission site. Please log into your account, locate the manuscript record, and check for the action link "View Attachments". If this link does not appear, there are no attachment files.]

While revising your submission, please upload your figure files to the Preflight Analysis and Conversion Engine (PACE) digital diagnostic tool,** [**https://pacev2.apexcovantage.com/**](https://nam10.safelinks.protection.outlook.com/?url=https%3A%2F%2Fpacev2.apexcovantage.com%2F&data=05%7C01%7Caamarillo%40javeriana.edu.co%7Ccd7ff305c0db40f994c208db873a9e2c%7Cdaf7990e8a3f409c9b762a5475098000%7C0%7C0%7C638252458087915807%7CUnknown%7CTWFpbGZsb3d8eyJWIjoiMC4wLjAwMDAiLCJQIjoiV2luMzIiLCJBTiI6Ik1haWwiLCJXVCI6Mn0%3D%7C3000%7C%7C%7C&sdata=%2Ba4%2FlU2q6znQ5WB6a24XmgtgQ0SZeX7X%2F3osnXrdBPE%3D&reserved=0)**. PACE helps ensure that figures meet PLOS requirements. To use PACE, you must first register as a user. Registration is free. Then, login and navigate to the UPLOAD tab, where you will find detailed instructions on how to use the tool. If you encounter any issues or have any questions when using PACE, please email PLOS at** [**figures@plos.org**](mailto:figures@plos.org)**. Please note that Supporting Information files do not need this step.**
